# Westminster Hospital

**Published:** 1829-11

**Authors:** 


### WESTMINSTER HOSPITAL.

### *Case of Diseased Testicle.*

In our last Number, page 303, we recorded a case of sarcocele, in which the use of bougies (which was recommended by Mr. Guthrie,) completely cured the disease, after various other means had been ineffectually tried by several eminent surgeons. In the following case, it is true, external irritation was produced by the tincture of iodine; but it is but fair to infer, with the previous case in our remembrance, that the use of the bougie was at least a very important auxiliary.

William Cox, aged fifty-three, has had an enlarged testicle since February last; applied at the Westminster Hospital on the 20th June, and was admitted under Mr. Guthrie. The right testicle is considerably enlarged, and some fluid can be distinctly perceived on the anterior part, covering an irregular hard testis. He complains of pain in the part, extending to the back. The cord appears to be sound.

Mr. Guthrie evacuated the fluid by means of a lancet and an eye probe, when the testicle became more distinct, very hard, knotted, irregular, and heavy, resembling very much a scirrhous testis, excepting that the pain was more constant, and less of a lancinating kind. The appearance of the man, and his state of health, were otherwise good.

Mr. Guthrie said that this was one of the many testicles that would have been immediately removed twenty years ago, but which was certainly curable. He directed him to take the submuriate of mercury, with the Ext. Conii, night and morning, until his mouth became sore; to have the testicle properly supported, and the hemlock poultice applied. Under this treatment he continued during the month of July: the testicle diminished in size, the hardness subsided, and the irregularities in a great measure disappeared.

The effects of the mercury on the mouth having gone off in August, Mr. Guthrie directed a bougie to be passed every second day, and the tincture of iodine to be applied to the scrotum in the following manner: The scrotum being shaved, a piece of lint, of a definite size, is to be dipped in the tincture, and applied to it, and to be changed for another piece, or to be moistened with the tincture when it becomes dry; and continued until it causes considerable irritation, which takes place in from twenty-four to forty-eight or sixty hours; at the end of which time the part to which the application has been made becomes hard, dry, and painful. A poultice is to be had recourse to for twenty-four hours, when the scale of cuticle comes off, leaving an irritable surface, very much like that of a blister. In fact, it is a different and elegant way of giving rise to a similar but more efficient irritation, and one to which the patient submits more readily than to a common blister.

Under this treatment, repeated from time to time, the patient has recovered: the pain is gone, the testicle is nearly of its natural size, and there remains only a small quantity of fluid to be absorbed.

---

